# Design and Performance Evaluation of a Novel Spiral Head-Stem Trunnion for Hip Implants Using Finite Element Analysis

**DOI:** 10.3390/ma16041466

**Published:** 2023-02-09

**Authors:** Md Mohiuddin Soliman, Muhammad E. H. Chowdhury, Mohammad Tariqul Islam, Farayi Musharavati, Sakib Mahmud, Muhammad Hafizh, Mohamed Arselene Ayari, Amith Khandakar, Mohammad Kaosar Alam, Erfan Zal Nezhad

**Affiliations:** 1 Department of Electrical, Electronic and Systems Engineering, Faculty of Engineering and Built Environment, Universiti Kebangsaan Malaysia, Bangi 43600, Selangor, Malaysia; 2 Department of Electrical Engineering, Qatar University, Doha 2713, Qatar; 3 Centre for Advanced Electronic and Communication Engineering, Department of Electrical, Electronic and Systems Engineering, Faculty of Engineering & Built Environment, Universiti Kebangsaan Malaysia (UKM), Bangi 43600, Selangor, Malaysia; 4 Department of Mechanical & Industrial Engineering, Qatar University, Doha 2713, Qatar; 5 Department of Civil and Architectural Engineering, Qatar University, Doha 2713, Qatar; 6 Department of Biomedical Engineering, University of Texas at San Antonio, San Antonio, TX 78249, USA

**Keywords:** spiral head-stem trunnion, contact pressure, sliding distance, wear rate

## Abstract

With an expectation of an increased number of revision surgeries and patients receiving orthopedic implants in the coming years, the focus of joint replacement research needs to be on improving the mechanical properties of implants. Head-stem trunnion fixation provides superior load support and implant stability. Fretting wear is formed at the trunnion because of the dynamic load activities of patients, and this eventually causes the total hip implant system to fail. To optimize the design, multiple experiments with various trunnion geometries have been performed by researchers to examine the wear rate and associated mechanical performance characteristics of the existing head-stem trunnion. The objective of this work is to quantify and evaluate the performance parameters of smooth and novel spiral head-stem trunnion types under dynamic loading situations. This study proposes a finite element method for estimating head-stem trunnion performance characteristics, namely contact pressure and sliding distance, for both trunnion types under walking and jogging dynamic loading conditions. The wear rate for both trunnion types was computed using the Archard wear model for a standard number of gait cycles. The experimental results indicated that the spiral trunnion with a uniform contact pressure distribution achieved more fixation than the smooth trunnion. However, the average contact pressure distribution was nearly the same for both trunnion types. The maximum and average sliding distances were both shorter for the spiral trunnion; hence, the summed sliding distance was approximately 10% shorter for spiral trunnions than that of the smooth trunnion over a complete gait cycle. Owing to a lower sliding ability, hip implants with spiral trunnions achieved more stability than those with smooth trunnions. The anticipated wear rate for spiral trunnions was 0.039 mm^3^, which was approximately 10% lower than the smooth trunnion wear rate of 0.048 mm^3^ per million loading cycles. The spiral trunnion achieved superior fixation stability with a shorter sliding distance and a lower wear rate than the smooth trunnion; therefore, the spiral trunnion can be recommended for future hip implant systems.

## 1. Introduction

Total hip replacement (THR) was the most common joint replacement in orthopedic surgery over the last two decades [1]. The National Joint Registry (NJR) documented 138,0472 hip replacements, of which 90% were initial replacements and 10% were revisions. In addition, 12.5% of implant revisions were performed due to implant joint failure due to wear [2]. In addition, metal ion-related complications, such as inflammatory reactions and pseudotumors, have been linked to the head-neck junction of hip implants [3,4,5,6]. Several aspects influence the functioning of the head-stem trunnion, including the angular discrepancy between the head and stem trunnion [7,8], the assembly process in the surgical intervention [9,10,11], trunnion geometry [12,13,14], acetabular head dimension [14,15,16,17], joint material combination [11,14,18], contact surface roughness [19,20,21] and possible contamination at the interface [22,23].

As a pre-clinical performance evaluation, the finite element model (FEM) analysis is required to validate the head-stem trunnion. To forecast various performance parameters and further improve trunnion design, researchers in the field of orthopedics have conducted computational FEM studies of implant design under real loading conditions. Ashkanfar et al. [24] studied the effects of microgroove tapered junction surfaces to evaluate the wear rate of the taper junction of a hip implant. A FEM analysis was performed on two hip implants with microgrooves and a smooth surface of tapering connection with a 36 mm CoCr femoral head. The analysis revealed that the wear rate of microgroove tapered connections was 1.4 times that of smooth tapered connections. Gustafson et al. [25] investigated the effects of microgroove surfaces on head-neck tapered contact mechanics using a well-established 12/14 head-neck tapered design with a CoCrMo femoral head taper and a Ti6Al4V stem taper. According to the finite element analysis (FEA), the microgroove tapered surface reduced the contact surface area, but increased contact pressures and plastic deformation. Chethan et al. [26] used FEM analysis to examine the effect of head-stem trunnion radius variation on the rate of wear formation at the hip implant joint. The wear rate increased as the proximal radius of the head-stem trunnion decreased, while the distal radius remained unchanged. Bitter et al. [27] and Vogel et al. [28] utilized FEM analysis to investigate the effect of angular mismatch on the sliding distance at the head-stem trunnion joint. The increased angular mismatch was found to result in greater micromotions at the joint interface. The impact of acetabular head size on the performance of the head-stem trunnion joint utilizing the FEM method has been reported in [14,15,16,17,29,30]. The reviewed studies indicated that a larger head diameter increased the sliding distance magnitude at the head-trunnion joint. Additionally, Tan et al. [13] analyzed 50 hip retrieval implants to determine the impact of taper surfaces on head-stem trunnion damage. Hip implants containing the 11/13 head-stem trunnion joint type caused the most damage compared to the 12/14 and 14/16 joint types, as reported by the author.

By analyzing recent studies on the performance of head-stem trunnions, it can be concluded that additional research is required to optimize the geometry of head-stem trunnions. This study undertook a comparative investigation of the performance of hip implants with smooth and spiral head-stem trunnions using the FEM method. The wear rate is regarded as the most important performance metric for comparing the two head-stem trunnion types. Contact pressure and sliding distance are estimated and analyzed as a function of wear estimation for both trunnion types under walking and jogging situations using finite element analysis. The wear rate was determined using contact pressure and sliding distance measurements. The retrieval experiments’ results further validate the performance of this wear rate. A particular head-stem trunnion type is recommended based on the findings of this study.

## 2. Materials and Methods

**Figure 1:**
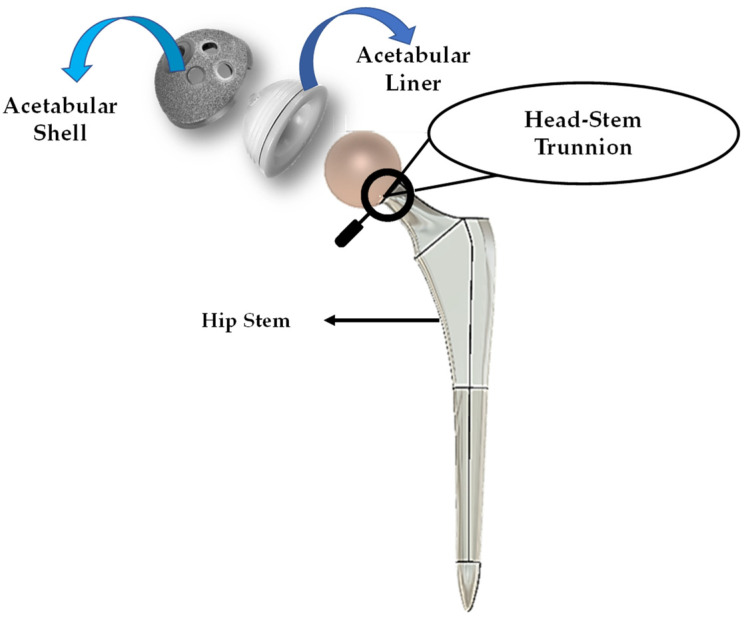
General view of a hip implant with components.

The head-stem trunnion model, a conventional hip implant and its components used in this study are shown in Figure 1. In the Section 2.1 below, the hip implant models with smooth and spiral head-neck taper junctions are shown with proper diagrams. Then, the finite element model with standard boundary and loading conditions is illustrated.

### 2.1. Modeling of Hip Implants

Since the number of hip replacement surgeries is constantly increasing [31,32], the production of hip implants is also increasing [33]. The SUMMIT tapered hip system has acquired worldwide recognition as an orthopedic hip implant during the past decade [34]. It is one of the most well-known hip implants manufactured by DePuy Synthes. The SUMMIT hip implant is considered clinically effective with a revision rate of 1.78% as per a total hip replacement (THR) study on 817 patients [35]. This includes both cemented and non-cemented solutions to accommodate a broad range of patient requirements [35]. After assessing these values, the SUMMIT Hip System was considered for this inquiry. Given that the hip implant’s dimensions rely on the patient’s bone structure, Wiki et al. [36] have showed that implant sizes 5 to 8 are most required for patients aged between 60 and 80. A large number of hip replacement procedures are performed for this age group [37]. The standard size 6 was considered for this investigation based on information provided by the implant manufacturer. First, a hip implant with smooth and spiral head-stem trunnions was designed. In both cases, the implant body dimension remained fixed. In this experiment, titanium alloy, Ti–6Al–4V, was selected for the stem material, and cobalt chromium alloy, Co–Cr–Mo, was selected for the acetabular head material because they are extensively used in the production of hip implant systems [34,38,39]. Later in this study, the proposed acetabular head-stem trunnion model will be verified for other material alloys. Table 1 summarizes the physical characteristics of material alloys evaluated in this study [40,41,42,43,44]. A 3D model of the hip implant was created using Fusion-360, a cloud-based 3D modeling software platform [45].

**Figure 2:**
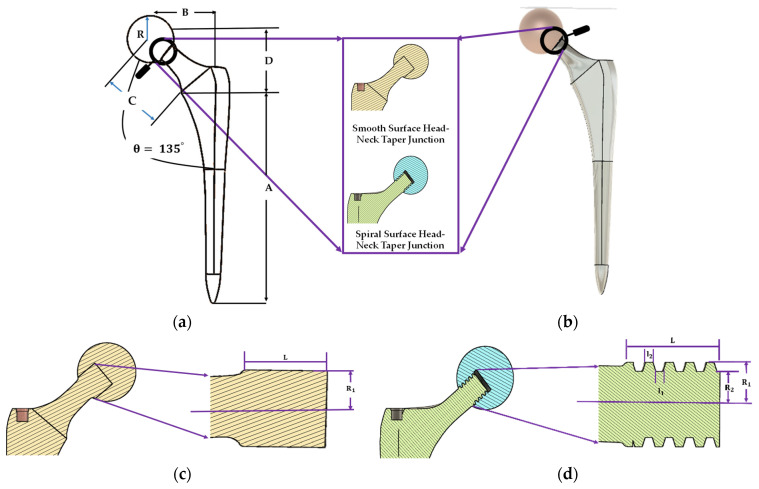
Illustration of a (**a**) 2D and (**b**) 3D geometrical view of the hip implant body and inner geometry of (**c**) smooth surface and (**d**) spiral surface of head-stem trunnion.

Fusion-360, a cloud-based 3D modelling, CAD, CAM and PCB software platform for product design and manufacturing, was utilized to build the 3D model in this study. The 2D geometry and 3D cad model view of the hip implant with smooth and spiral surface head-stem trunnion designed in Fusion 360 software is depicted in Figure 2. Table 2 contains geometrical parameter values of the hip implant body and head-stem trunnion joint.

### 2.2. Boundary and Loading Conditions with the Finite Element Model Framework

Boundary conditions are the standards which are used for evaluating the mechanical performance of the implant. In this study, boundary conditions included the implant’s fixed support zone and loading conditions for estimating mechanical performance using a computational finite element model. This study follows ISO-7206-6:2013 standard for building up a mechanical fatigue testing system for hip implant head-stem trunnion joints in computational model environments. The stem is fastened to the distal neck, as reported in [46,47,48,49].

**Figure 3:**
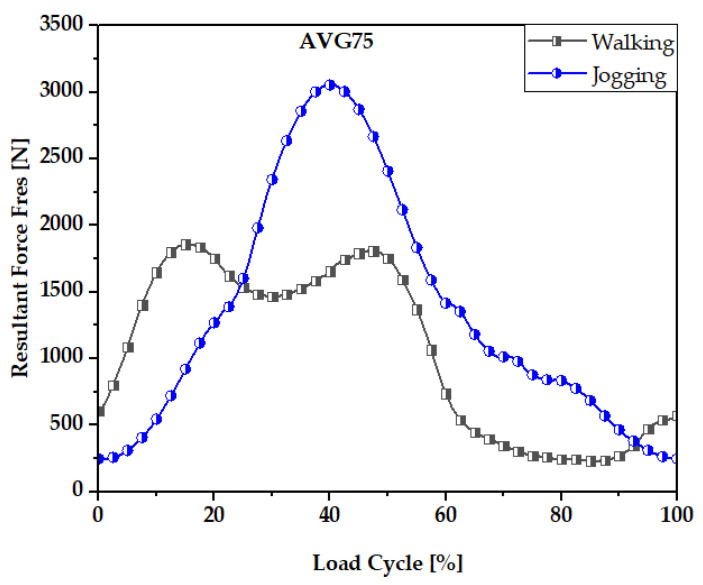
Resultant Force, F_res_, values for walking and jogging loading conditions.

Many in vitro [50,51,52,53] FEM [7,46,54] investigations have been undertaken to understand fretting wear damage to head and stem materials in terms of assembly loads. Many investigations questioned the accuracy of simplified in vitro tests compared to a real mechanical environment which is present for many real human activities. Precise loading is needed for reliable FEM data. The mechanical performance and wear rate of the suggested hip implant model were based on normalized human activities. This study incorporated force data from the most physically demanding everyday activities (walking and jogging) published by Bergmann et al. [55]. Walking is the most common activity, whereas jogging is one of the most physically demanding activities. Walking and jogging loads from 50 to 68-year-old subjects weighing from 70 to 120 kg (AVG 75 kg) were used here. In [52], the mechanical characterization of hip implant functional loading conditions has been presented. Each measurement’s details are documented on OrthoLoad.com [56]. The extensive information for each measurement was documented and is accessible via the publicly available repository at www.OrthoLoad.com. Figure 3 shows the walking and jogging resultant force, $F_{\mathrm{res}}$. For 10% to 55% of each gait cycle, walking required more force while this was between 25% and 55% for jogging.

**Figure 4:**
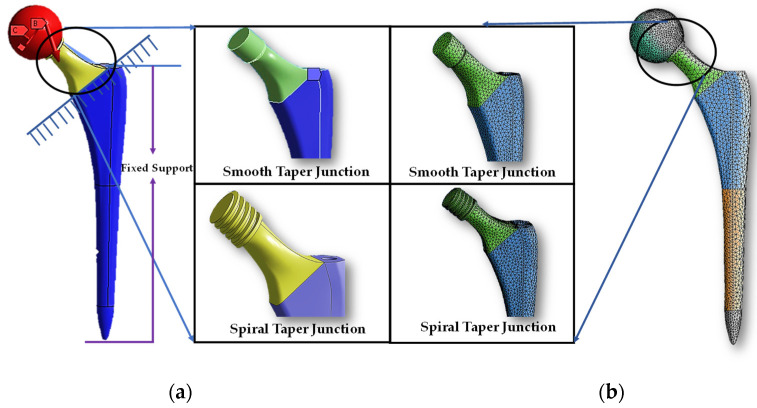
(**a**) Three-dimensional finite element model view in Ansys workbench interface and (**b**) meshing structure of a hip implant with smooth and spiral head-stem trunnions.

**Figure 5:**
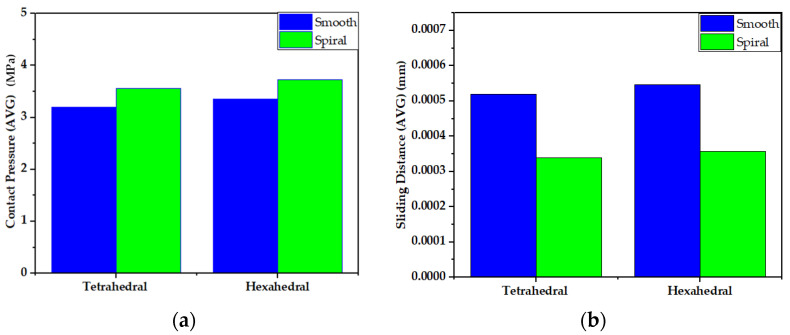
Impact of mesh element variation on (**a**) average contact pressure and (**b**) average sliding distance for both head-stem trunnion types.

**Figure 6:**
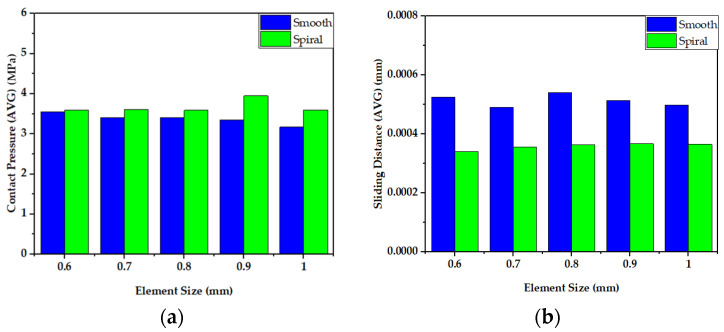
Impact of mesh element size variation on (**a**) average contact pressure and (**b**) average sliding distance for both head-stem trunnion types.

The wear rate and mechanical performance of hip implants with smooth and spiral taper junctions were estimated using a computational finite element model computational analysis. In this work, Ansys 2021 (Ansys Inc., Canonsburg, PA, USA) was used to perform FEM analysis on the hip implant with varied taper junction geometry. The 3D geometry was created in Fusion 360 (Autodesk) and it was imported into Ansys 2021. Each hip implant design uses ISO-7206-4-2013-compliant FEM boundary conditions. The implant’s acetabular head received the stresses and moments applied to it. The acetabular head is dynamically loaded with forces and moments during walking. These dynamic loads can be considered 600 static loads. Computer-based FEM analysis requires adequate meshing. The tapered junction’s contact surface is frictional, and a 0.2 friction coefficient was used [7,57]. Titanium and cobalt chrome materials have a friction coefficient of 0.15 to 0.4 [58,59]. More details about meshing selection are illustrated with performance analysis. Figure 4 shows a three-dimensional finite element model of a hip implant with smooth and spiral head-stem trunnions in Ansys Workbench 2022. Based on comparative performance experiments employing mesh sizes of 0.6–1.0 mm, this research selected 10 nodes of quadratic tetrahedral mesh elements C3D10 of 1.0 mm. There are four distinct types of mesh elements: linear tetrahedral, quadratic tetrahedral, linear hexahedral and quadratic hexahedral. A quadratic mesh element employs a non-linear shape function in which nodes are interpolated using a polynomial of a higher order [60]. Recent research into hip implant-based finite element studies have used quadratic tetrahedral [7,61,62,63] mesh elements more than any other form. According to Ansys [64], the preferred choice for complicated nonlinear geometry is tetrahedral mesh elements. Automatic mesh creation in the hexahedral mesh approach is computationally infeasible in terms of nonlinear complicated geometry [65]. In addition, a preliminary finite element analysis was conducted to show the impact of mesh element variation on average contact pressure and average sliding distance for both head-stem trunnion types, as shown in Figure 5. With mesh elements varying from tetrahedral to hexahedral, the average contact pressure and sliding distance vary by around 5%. Nevertheless, the experimental simulation time for hexahedral is more than twice that of tetrahedral mesh elements. Hence, to prevent experimental complexity and based on suggestions in the literature, we have considered tetrahedral mesh elements for this research. Moreover, Figure 6 shows how mesh size affects contact pressure and sliding distance. Here, the highest point of walking load was used as the load value and CoCr–Ti6Al4V was chosen for the head-stem trunnion because it is extensively used in the production of hip implant systems [34,38,39]. With mesh element sizes ranging from 0.6 to 1 mm, there was a small difference of less than 5% in the average contact pressure and sliding distance. Hence, an element size of 1 mm was considered to avoid longer simulation times. Notably, the mesh element numbers are 49,226 and 56,362, while the mesh node numbers are 85,585 and 97,762 for smooth and spiral head-neck trunnion design, respectively.

### 2.3. Wear Estimation and Associated Performance Parameters Theory

Wear studies are important in determining joint performance. Earlier works [46,61,66,67,68] have utilized the Archard wear model to estimate the wear rate according to Equation (1):

(1) $$dV_{w}=\Delta AdH=K_{w}*p*\Delta A*dS\;$$

Here,

(2) $$dH=K_{w}*p*dS\;$$

where dH is the wear depth (mm/cycle), p is the contact pressure, dS is the sliding distance and ΔA is the contact area (mm^2^). Wear coefficient, $K_{w}$, is the wear constant for different separate biomaterials. Table 3 contains the wear coefficient utilized in this study.

Integrating Equation (2) over a complete gait cycle and time, t, and calculating the wear depth at any point [62] gives:

(3) $$H=K_{w}\int_{0}^{t}p*\mathrm{dS}$$

here, the sliding distance, dS, at a single point is estimated by summing the sliding distance value over the whole gait cycle. Contact pressure, p, can be taken as a time instance, t, value over the whole gait cycle. The volume of wear on the joint surface can be estimated by adding up the area of contact A.

(4) $$\left(t\right)=n*K_{w}\int_{0}^{A}\left(\int_{0}^{t}p*dS\right)dA$$

here, contact pressure, p, is taken as the average value over joint surface area, A, and the complete gait cycle. Here, dS is the average sliding distance value over contact surface, A, at time, t. Hence, the total sliding distance value can be estimated by Equation (5). Here, time t allotment is related to the respective loading condition applied.

$$\Delta dS\left(t\right)=\left|dS\left(t\right)-dS\left(t+t_{i}\right)\right|$$

(5) $$Summed\;Sliding\;Distance=\overset{t=Ns}{\underset{0}{\sum }}\Delta dS\left(t\right)$$

The results of the sliding distance and contact pressure are influenced by stem material properties, which can be obtained using Ansys software’s FEM analysis. In this study, the rate of wear was estimated following non-adaptive finite element studies found in the review article [58]. Contact pressure and sliding distance were obtained from the finite element model in Ansys simulating software over a gait cycle, and wear was calculated using the Archard wear model for the required number of cycles. There are four other wear estimation models, namely the Saikko model, the Liu model, the Kang model and the Petrella model [69]. These wear models consider the cross shear ratio (CSR) instead of the wear coefficient [69,70] and are mostly utilized for estimating wear on the acetabular head-polyethene liner. Additionally, the CSR value for metal-on-metal (MoM) material combinations is still under research. Hence, owing to the unavailability of CSR data for MoM material combinations, we mostly utilized the Archard wear model.

## 3. Results Evaluation and Discussion

Firstly, the mechanical performance parameters obtained from FEM analysis, the contact pressure and sliding distance for both types of trunnion for walking and jogging loading conditions are illustrated. Then, using the described wear model, the wear generation rates from both trunnion types are estimated for a standard number of cycles for the two loading conditions. Finally, the estimated wear data using a finite element model is validated in association with the data collected from the in vivo study.

### 3.1. Contact Pressure Distributions

**Figure 7:**
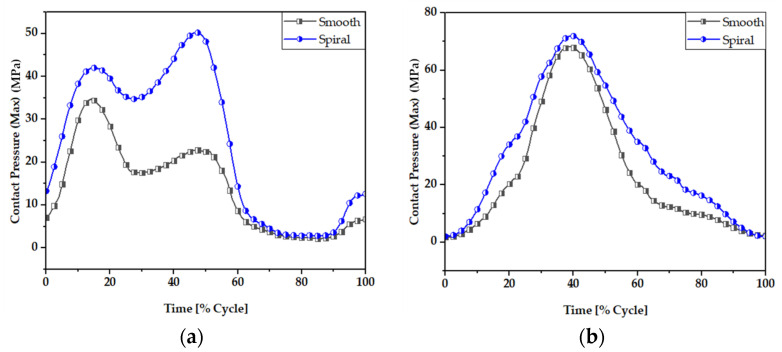
Maximum contact pressure (MPa) of smooth and spiral head-neck tapered joints during (**a**) walking and (**b**) jogging loading conditions.

**Figure 8:**
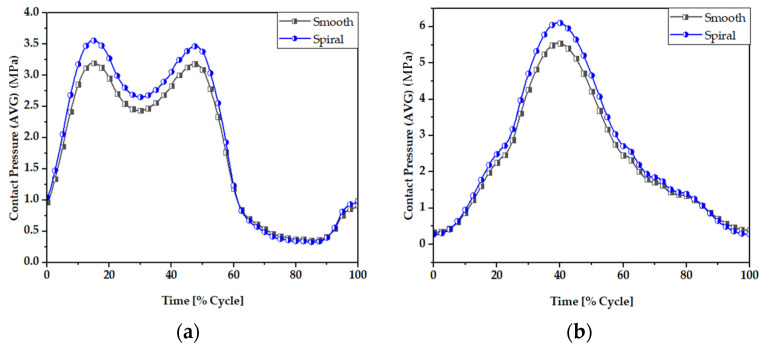
Average contact pressure (MPa) of smooth and spiral head-neck tapered joints during (**a**) walking and (**b**) jogging loading conditions.

Contact pressure is the fundamental parameter for predicting the wear generation rate in hip implant joints following wear estimation theories. By studying the contact pressure distribution inside a joint, the potential contact damage can be estimated. Figure 7 and Figure 8 illustrate the comparative view of maximum and average contact pressure of smooth and spiral head-neck tapering joints over walking and jogging loading conditions, respectively. Here, the maximum and average contact pressures were determined for complete gait cycles using 600 samples for each activity. The average contact pressure was calculated by dividing the total contact pressure by the contact area of the corresponding trunnion joints. Notably, under each loading situation, the spiral tapered design achieved a higher maximum contact pressure than the smooth trunnion joint in a specific contact region. While jogging resulted in higher maximum and average contact pressures, walking loading conditions resulted in lower maximum and average contact pressure values. Hence, it can be said that the highest force loading condition (jogging) obtained higher contact pressure in both joint types. A similar performance was found in [46], where authors reported that contact pressure is directly related to force imposed on the hip implant system. Interestingly, the average contact pressure value for the proposed spiral tapered design was only slightly greater than that of the smooth tapered junction in all loading conditions. This is owing to the uniform contact pressure distribution on the spiral trunnion contact area, even though a smaller contact area results in a higher maximum contact pressure than in the smooth trunnion.

**Figure 9:**
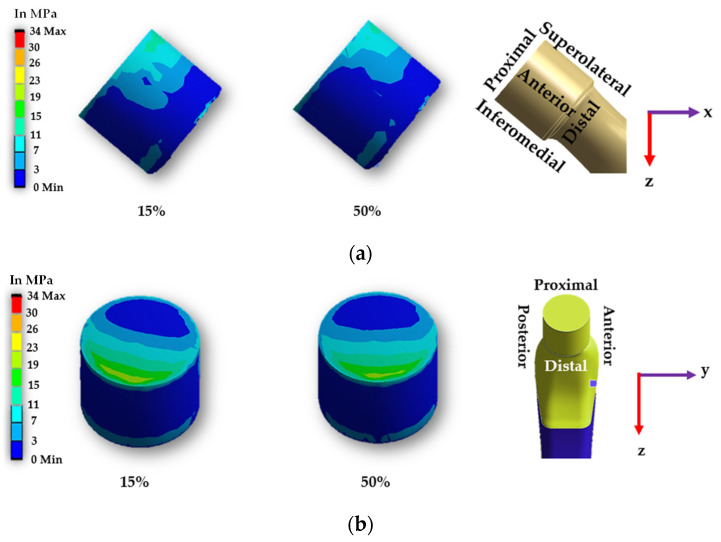
Contact pressure distribution over a smooth head-neck tapered joint from the (**a**) front and (**b**) right side during 15% and 50% of walking loading condition gait cycle.

**Figure 10:**
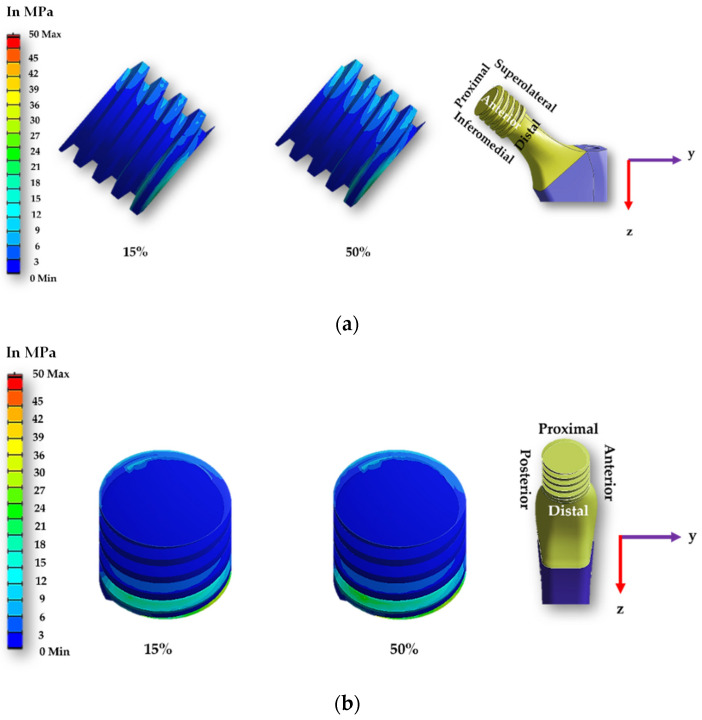
Contact pressure distribution over a spiral head-neck tapered joint from the (**a**) front and (**b**) right side for 15% and 50% of walking loading condition gait cycle.

Figure 9 and Figure 10 display the contact pressure distribution in the contact area of smooth and spiral head-neck tapered joints, respectively, for walking loading conditions. The contact pressure distribution is shown for 15% and 50% of walking and 50% of jogging loading conditions gait cycle, as these are the highest load points according to Figure 2. Figure 9 and Figure 10a,b show the distribution of contact pressure over the smooth and spiral head-neck tapered junction from the right and front views, respectively, for the walking load condition. As the contact pressure is exactly opposite the head and stem taper surfaces, the variability in contact pressure is depicted in Figure 9 and Figure 10 as spread across both the head and neck tapered surfaces.

In the front view of the junction depicted in Figure 9 and Figure 10, the contact pressure is concentrated mostly in the top of the super-lateral and inferomedial distal portion of both joint types during 15% and 50% of the walking loading gait cycle. Additionally, the right-side view depicts that the proximal part and inferomedial distal part of the junction have higher contact pressure for the smooth tapered joint. As illustrated in Figure 9b, the greatest contact pressure of 34.67 MPa was found in the proximal area of the smooth head-neck joint during 15% of the gait cycle under walking loading conditions, where a peak load of 1830 N is applied. In the case of the smooth trunnion, the proximal segment concentrates more contact pressure.

In contrast, the superolateral distal area had a lower contact pressure due to its weaker contact state. In the spiral surface, the inferomedial distal area had the maximum contact pressure, whereas the superolateral region had consistent contact pressure. Due to the strong contact status in these places, contact pressure in the proximal region was lower. As illustrated in Figure 10b, the greatest contact pressure of 50.285 MPa was found in the inferomedial distal section for the spiral head-neck joint during about 50% of the gait cycle under walking loading conditions, where a peak load of 1800 N was applied. The distribution of contact pressure over a smooth and spiral head-neck tapered junction for walking and jogging loading scenarios with a complete gait cycle is depicted in Supplementary Figures S1–S4. Contact pressure distribution is shown by the front and right-side view for both joints. According to an inspection of the front and right-side view for all loading situations, the contact pressure is mainly concentrated in the top of super lateral, proximal and inferomedial distal regions of the smooth head-neck tapered junction over the gait cycle of the respective loading condition. While for the spiral joint, the contact pressure is mainly concentrated in the top of superolateral and inferomedial distal regions of the spiral head-neck tapered junction over the gait cycle of the respective loading condition. Even though the contact pressure distribution position across smooth and spiral head-neck tapering junctions is almost uniform for all loading circumstances, the contact pressure concentration value varies under different loading conditions with different gait cycle phases.

It should be noted that the spiral shape trunnion influences the distribution and fixing of contact pressure. In the case of the spiral trunnion, nearly homogeneous contact pressure throughout the superolateral area produced a stronger fixation. While in the event of a smooth contact, the contact pressure is mostly concentrated in the proximal and proximal superolateral regions; there is no uniform distribution of contact pressure. Owing to the spiral design, the fixation status in the trunnion contact region is significantly improved compared to smooth trunnions. A discussion on sliding distance will be presented to give a clearer overview of the fixation ability for both trunnion cases.

### 3.2. Sliding Distance

**Figure 11:**
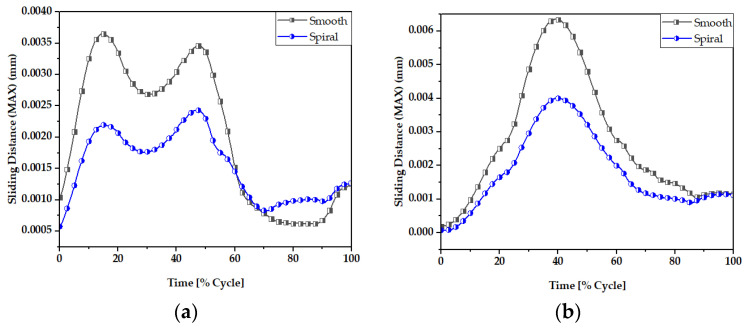
Maximum sliding distance of smooth and spiral head-neck tapered joints during (**a**) walking and (**b**) jogging loading conditions.

**Figure 12:**
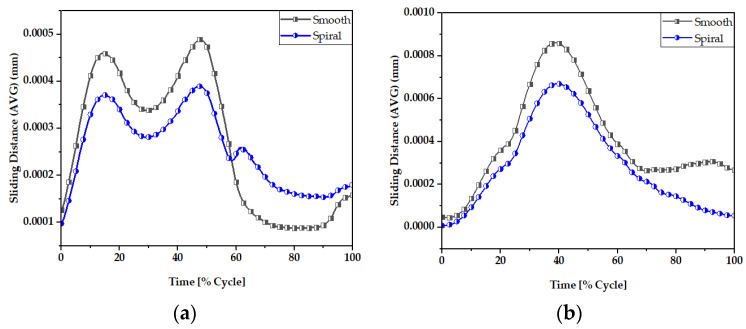
Average sliding distance (mm) of (**a**) smooth and (**b**) spiral head-neck tapered joints.

**Figure 13:**
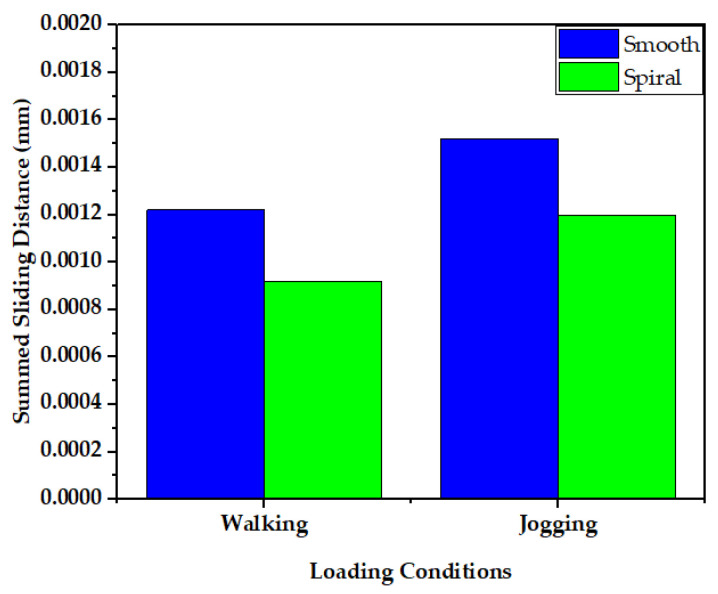
Comparison of sliding distance of smooth and spiral head-neck tapered joints during walking and jogging loading conditions.

The sliding distance at the head-neck junction interface has a key role in defining the functioning of the junction, as it can contribute to wear rate [66,67] and the subsequent release of material debris into the surrounding tissues. Figure 11 and Figure 12 illustrate the maximum and average sliding distance, respectively, of smooth and spiral head-neck tapering joints for walking and jogging loading conditions. Here, the maximum and average sliding distances were determined for complete gait cycles using 600 samples for each activity. The average sliding was calculated by dividing the total sliding distance by the contact area of the corresponding tapered joints. Hence, the measurement procedure was performed in similar way to the contact pressure measurement. Notably, the spiral tapered design achieved a shorter sliding distance under each loading condition than the smooth tapered junction. Here, jogging resulted in the highest maximum and average sliding distance values for both joint cases compared to walking. Therefore, the maximum force loading condition (jogging) resulted in a greater sliding distance for both joint types. Similar findings were discovered in [46], where it was found that the micro-motion achieved maximum values for the stair-up, stair-down and one-leg standing conditions. The spiral geometrical shape forms a stronger mechanical bond in the head-neck tapered joint that causes a lower sliding distance compared to the smooth joint. Figure 13 depicts that the sliding distance was reduced by 25% for spiral joints compared with the smooth joint type under both loading conditions. The sliding distance found for a smooth head-stem trunnion can be confirmed by an experiment in [71], where the sliding distance was found to be 0.0016±0.0006 for a load of 0.3–2.3 kN, while in this FE study, a smooth joint during walking (1.9 kN load) was found to have a sliding distance of 0.0012 mm, which is very close to the experimental value found in the cited study.

**Figure 14:**
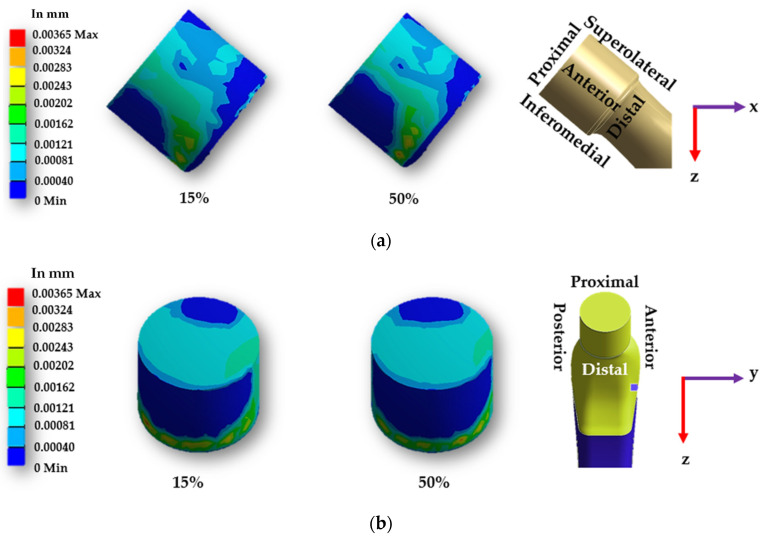
Sliding distance map over a smooth head-neck tapered joint from the (**a**) front and (**b**) right side for the walking loading condition.

**Figure 15:**
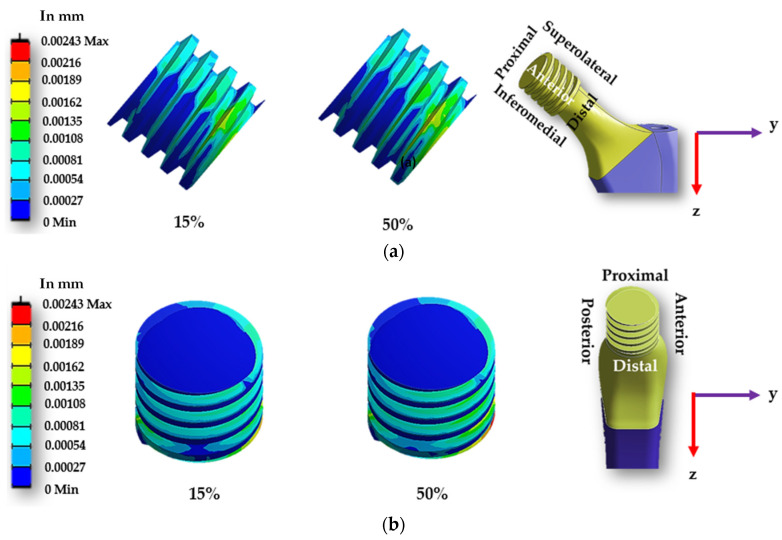
Sliding distance map over a spiral head-neck tapered joint from the (**a**) front and (**b**) right side for the walking loading condition.

Figure 14 and Figure 15 display the sliding distribution in the contact area of smooth and spiral head-neck tapered joints for walking loading conditions at 15% and 50% of gait cycle instances. In the front view and right-side view of the junction depicted in Figure 14a,b, the sliding distance is concentrated mostly in the top of the superolateral, inferomedial distal and anterior portion of smooth joint type during 15% and 50% of the walking loading gait cycle. At 15% of the gait cycle, the inferomedial-distal part of the smooth joint had the highest sliding distance, at 0.00365 mm. In the case of the spiral joint case, the sliding distance uniformly occurred in all directions of the contact surface except the proximal surface. While the maximum highest sliding distance of 0.00243 mm over the gait cycle occurred in the anterior distal section, which was lower than the maximum of the smooth joint type.

Supplementary Figures S5–S8 show the sliding distance over the smooth and spiral head-neck tapered junctions for walking and jogging loading scenarios with a full gait cycle. For both joints, the front and right-side views show sliding distance distribution. From the front and right-side views, it appears that most of the sliding distance occurs in the superolateral, proximal, anterior and inferomedial distal regions of the smooth head-neck tapered junction during the gait cycle of each load condition. While for the spiral joint, the sliding distance is mostly distributed over all regions except for the proximal of the spiral head-neck tapered junction over the gait cycle of the respective loading conditions. In this instance, the spiral design generated a greater contact pressure in the superolateral position, resulting in an increased fixation stability in the superolateral, inferomedial and anterior parts. Due to a more rigid attachment in the superolateral region, the sliding distance in the proximal region was close to zero, but it was also lower on the other sides. Hence, it can be said that the hip implant with a spiral trunnion will be more mechanically stable than a hip implant with a smooth trunnion.

Notably, the sliding distance is lower in the spiral joint case for both loading conditions compared to the smooth joint case. Therefore, based on theories on estimating wear, a hip implant with a spiral head-stem trunnion will generate less wear than one with a smooth trunnion.

### 3.3. Wear Rate Estimation and Validation

**Figure 16:**
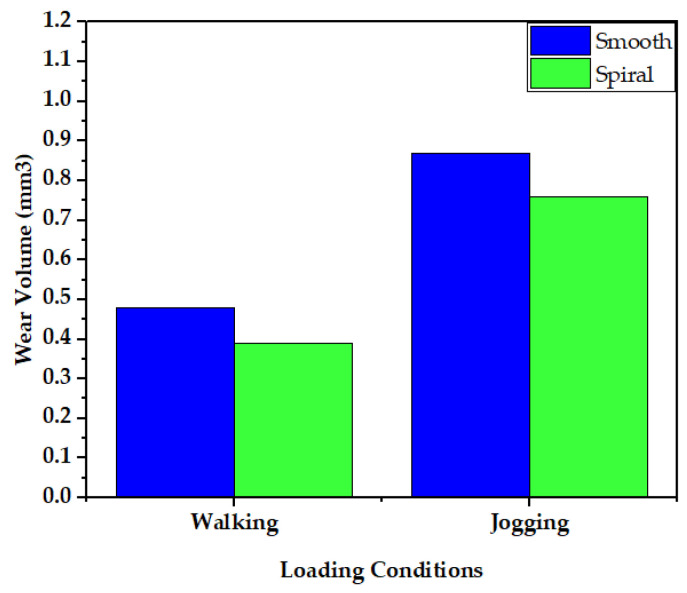
Comparison of wear generation rates in smooth and spiral head-neck tapered joints utilizing the Archard wear estimation model.

Figure 16 shows a comparison of the wear rate in smooth and spiral head-stem trunnions using the Archard and Saikko wear estimation models. The results in Figure 16 illustrate that a hip implant with a smooth trunnion produced a wear volume of 0.48 mm^3^, while for a spiral joint this value was 0.39 mm^3^ during walking loading conditions for 10 million gait cycles, which is about 10% less than the smooth joint. Similarly, the smooth joint produced a wear volume of 0.87 mm^3^, while a spiral joint produced a value of 0.76 mm^3^ during walking loading conditions for 10 million gait cycles, which is also about 10% less than the smooth joint.

The wear fraction can be used to estimate how much material has been worn away from each surface of the head-stem joint. A wear fraction of (1:0) means that all wear was removed from one joint surface, either the head or the stem. A wear fraction of (0.5:0.5) would imply that wear was removed in the same amount from both contact joint surfaces. The wear fractions correlated with CoCrMo–Ti6Al4V as a head-stem material combination was calculated as (0.9:0.1) following the work of [65]. Table 4 contains data on the wear rate on the head taper and stem trunnion contact surface.

The estimated wear rate of the smooth trunnion case in this study can be verified by experimental data from the retrieval of hip implant data from previous years. Since the retrieved data described the smooth joint type forms of hip implant trunnions, the smooth trunnion wear rate was addressed during validation. A wear performance report of hip implants was given in [66], where data were obtained from retrieved hip implants. The study examined the wear data of 92 implants with a tapered head-stem joint made of a CoCr–Ti material combination. This study found that the average rate of wear at the head-stem tapered joint was 0.19 mm^3^/year (range: 0.03–0.75), and the mean duration between implant substitutions was 50 months. Quantitative studies [67,68] of human activity after total hip replacement showed that people with hip replacements usually take at least 5000 steps a day. Therefore, it can be estimated that each hip implant joint will go through about 1 million cycles each year. Langton et al. [6] reported the wear performance from a retrieved hip implant system, where data were examined from 111 retrieved hip implants. This study reported a wear rate of 0.127 mm^3^/year (range 0.01 to 3.15) for the Articuleze acetabular head and Depuy titanium stem tapered joint, and the implant revision time was 1 to 5 years. The estimated wear rate for the hip implant with the smooth head-stem tapered joint was 0.048 mm^3^ per year for walking loading conditions, which is in the range of the measured wear rate as reported in [6,66]. The approximate similarity between the findings of this study and measured wear degradation of retrieved hip implants validates the 3D FE model, the loading and boundary conditions and the wear model utilized in this investigation. Notably, the spiral trunnion joint has a lower wear rate than the smooth trunnion joint under both loading conditions. Consequently, spiral trunnion hip implants will be more mechanically stable than those with smooth trunnions.

## 4. Conclusions

A comparative analysis of computed contact pressures, sliding distances and wear rates on smooth and spiral head-stem trunnions of hip implants utilizing the FEM method is presented. This inquiry is conducted for the load cases of walking and jogging to comprehend the effects of dynamic loading situations. The results of the study indicate that the spiral head-stem trunnion has a stronger fixation stability with a shorter sliding distance than the smooth trunnion, while the average contact pressure is nearly identical in both situations. In addition, the anticipated rate of wear production is 0.039 mm^3^ for spiral trunnions which is approximately 10% lower than that for smooth trunnions (wear rate of 0.048 mm^3^ per million cycles). The estimated wear rate in this study is verified by experimental data from retrieved hip implants. Based on the findings of this study, a hip implants with spiral trunnions can be an effective means of optimizing hip implant systems.

## Figures and Tables

**Table 1:** Physical features of material alloys considered in this investigation [40,41,42,43,44].

	Elastic Modulus (GPA)	Yield Strength (MPa)	Ultimate Strength (MPa)	Poisson Ratio	Density (kg/m^3^)
Ti6Al4V	110	910	1000	0.342	4400
Co–Cr	220	840	1280	0.3	8500

**Table 2:** Geometrical parameters values of the hip implant body and head-stem trunnion.

Parameter	Values (mm)	Parameter	Values mm
Stem Length—A	150	Neck Trunnion Length—L	12
Neck Length—B	33.5	Channel Thickness—$l_{1}$	0.85
Offset—C	35.9	Flight Thickness—$l_{2}$	0.89
Leg Length Adjustment—D	43.2	Neck Trunnion Radius—$R_{1}$	6.4
Head Radius—R	32	Channel Radius—$R_{2}$	5.025

**Table 3:** Wear coefficient, $K_{w}$, for different material combinations.

Material Combinations	$\mathrm{Wear}\;\mathrm{Coefficient},\;K_{w}$ $,\;\;MP^{-1}$	Reference
CoCr–Ti6Al4V	$2.97\times 10^{-8}$	[54]

**Table 4:** Wear rate on head taper and stem trunnion contact surface.

Joint Type	Loading Condition	Wear Volume (mm^3^)		
		Head Taper	Stem Trunnion	Total
Smooth	Walking	0.432	0.048	0.48
	Jogging	0.72	0.08	0.8
Spiral	Walking	0.351	0.0351	0.39
	Jogging	0.621	0.069	0.69

## Data Availability

Not applicable.

## Supplementary Materials

The following supporting information can be downloaded at: https://www.mdpi.com/article/10.3390/ma16041466/s1, Figure S1: Contact Pressure Distribution Over Smooth Head-Neck Tapered Joint in, (a) Front and (b) Right Side View for Walking Loading Condition; Figure S2: Contact Pressure Distribution Over Smooth Head-Neck Tapered Joint in, (a) Front and (b) Right Side View for Jogging Loading Condition; Figure S3: Contact Pressure Distribution Over Spiral Head-Neck Tapered Joint in, (a) Front and (b) Right Side View for Walking Loading Condition; Figure S4: Contact Pressure Distribution Over Spiral Head-Neck Tapered Joint in, (a) Front and (b) Right Side View for Jogging Loading Condition; Figure S5: Sliding Distance Distribution Over Smooth Head-Neck Tapered Joint in, (a) Front and (b) Right Side View for Walking Loading Condition; Figure S6: Sliding Distance Distribution Over Smooth Head-Neck Tapered Joint in, (a) Front and (b) Right Side View for Jogging Loading Condition; Figure S7: Sliding Distance Distribution Over Spiral Head-Neck Tapered Joint in, (a) Front and (b) Right Side View for Walking Loading Condition; Figure S8: Sliding Distance Distribution Over Spiral Head-Neck Tapered Joint in, (a) Front and (b) Right Side View for Walking Loading Condition.
