# Supplementary material for: Design and Performance Evaluation of a Novel Spiral Head-Stem Trunnion for Hip Implants Using Finite Element Analysis
Source: Materials (Basel). 2023 Feb 9;16(4):1466. doi: 10.3390/ma16041466 (PMC9962303; doi:10.3390/ma16041466)
Supplement: Supplementary file 1 [file materials-16-01466-s001.zip › materials-2015141-supplementary.pdf]

Supplementary Document

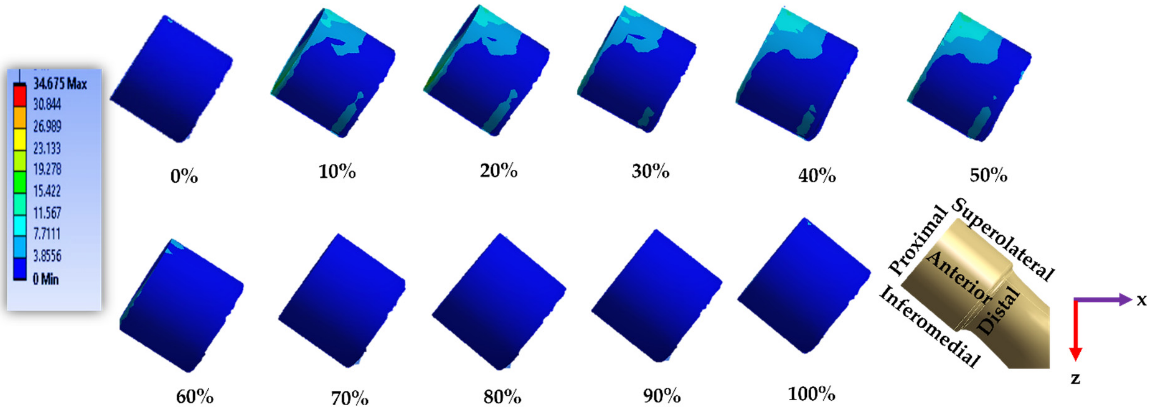

(a)

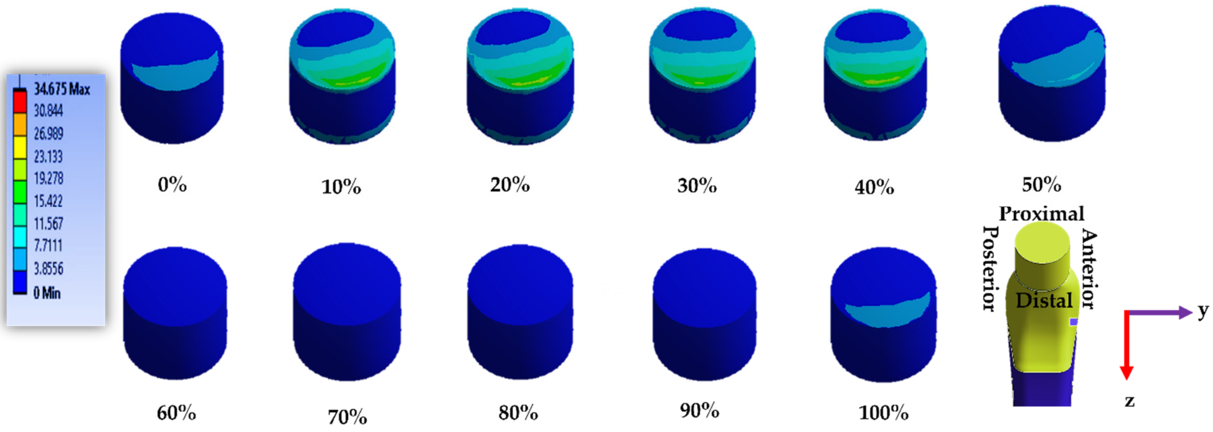

(b)

Figure S1. Contact Pressure Distribution Over Smooth Head-Neck Tapered Joint in, (a) Front and (b) Right Side View for Walking Loading Condition.

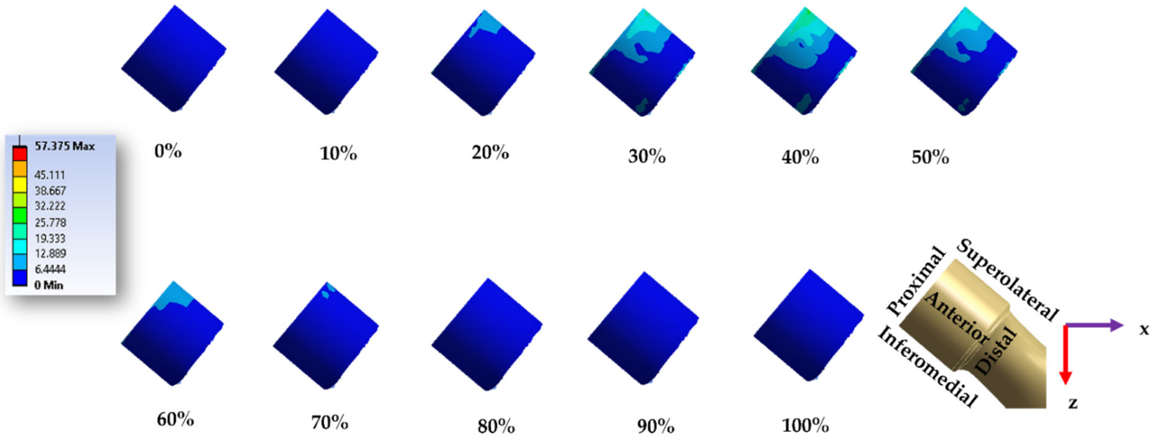

(a)

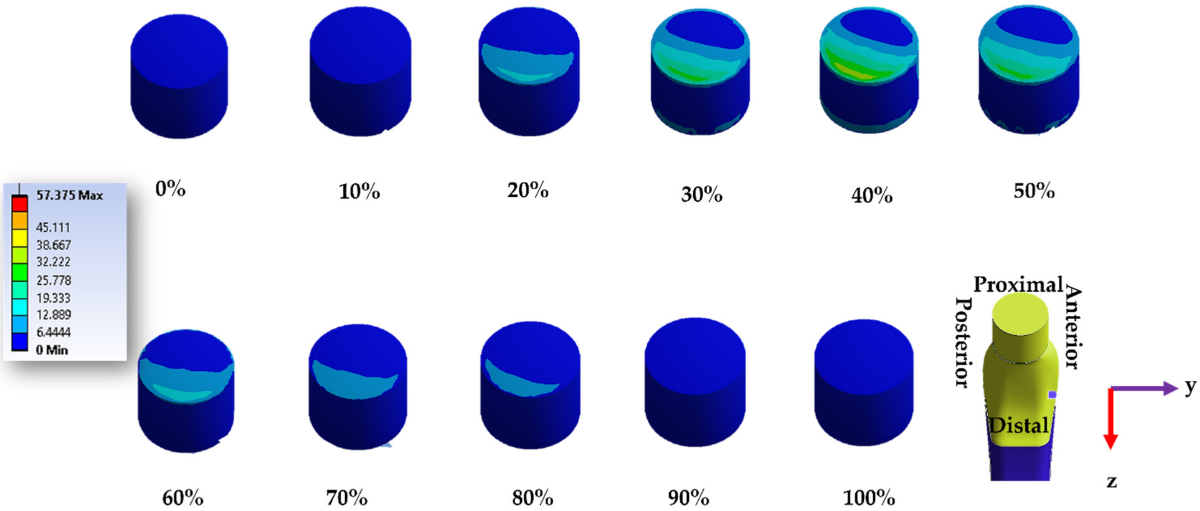

(b)

Figure S2. Contact Pressure Distribution Over Smooth Head-Neck Tapered Joint in, (a) Front and (b) Right Side View for Jogging Loading Condition.

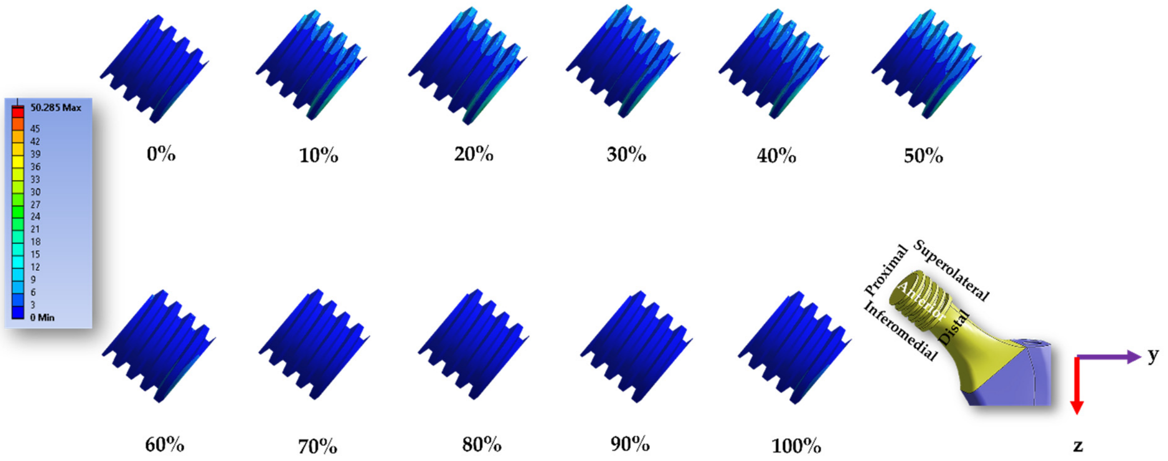

(a)

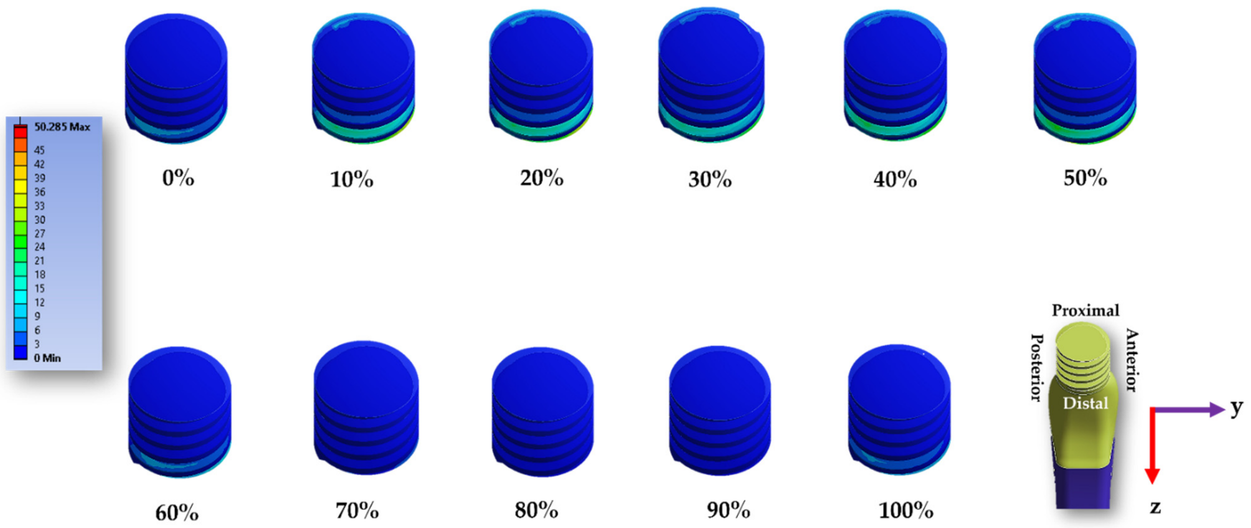

(b)

Figure S3. Contact Pressure Distribution Over Spiral Head-Neck Tapered Joint in, (a) Front and (b) Right Side View for Walking Loading Condition.

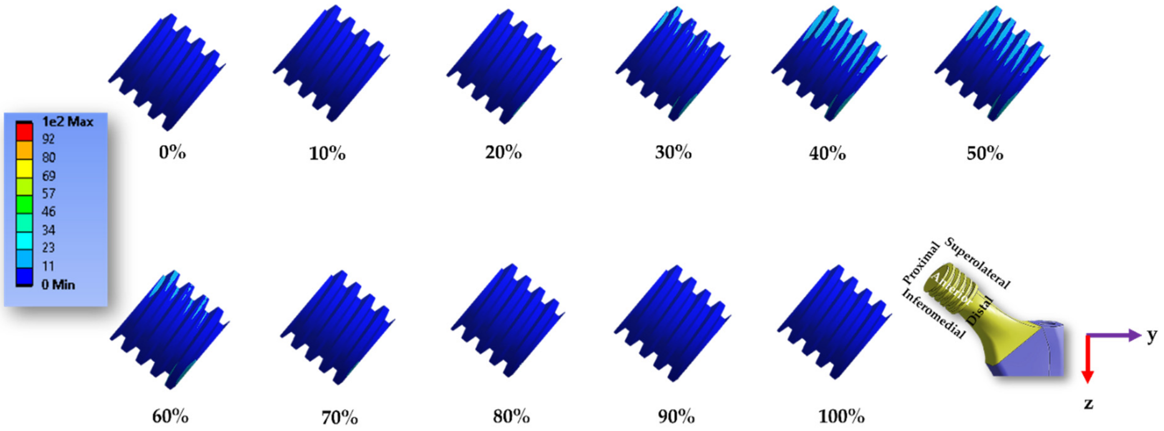

(a)

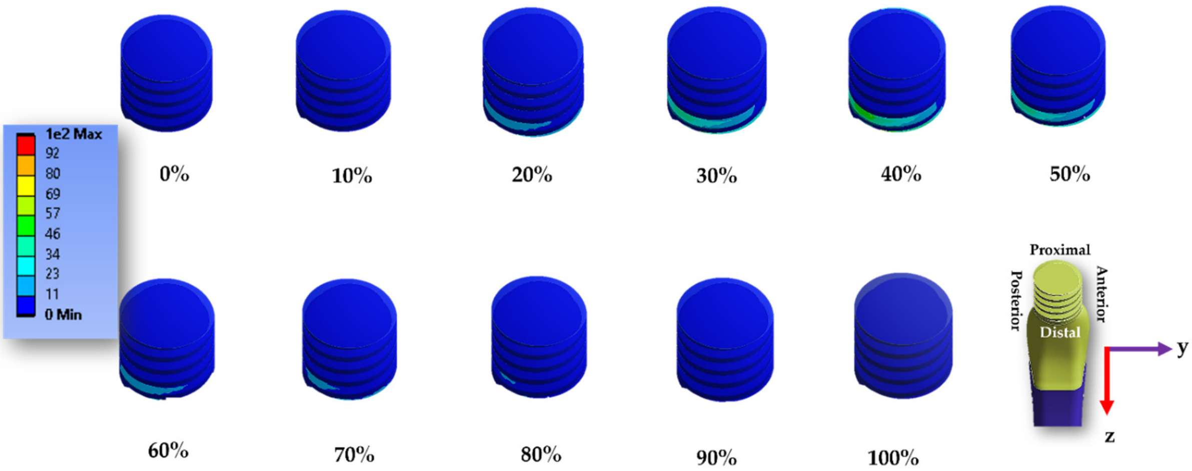

(b)

Figure S4. Contact Pressure Distribution Over Spiral Head-Neck Tapered Joint in, (a) Front and (b) Right Side View for Jogging Loading Condition.

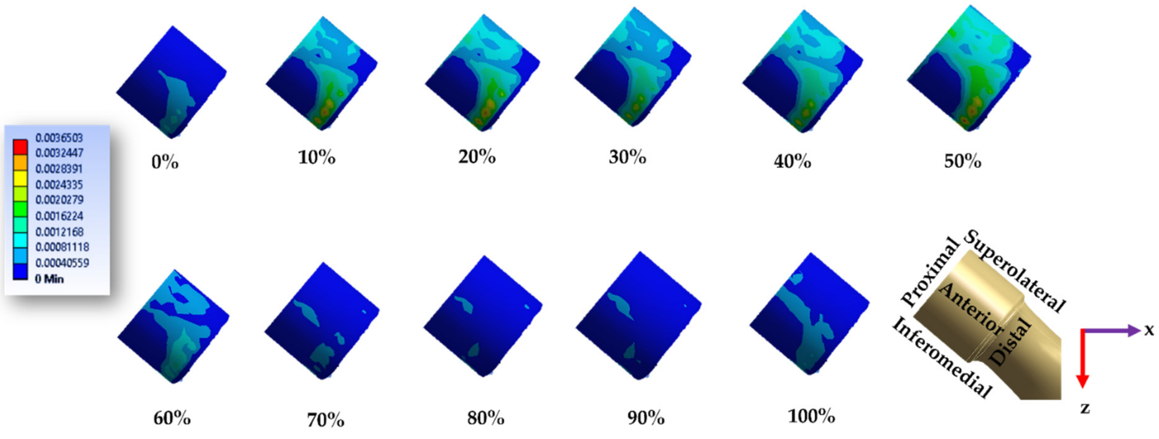

(a)

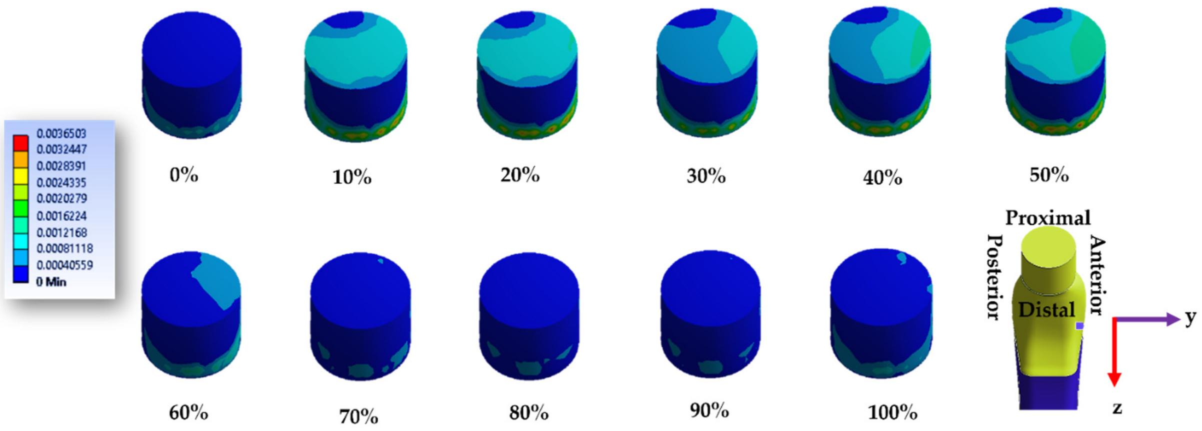

(b)

Figure S5. Sliding Distance Distribution Over Smooth Head-Neck Tapered Joint in, (a) Front and (b) Right Side View for Walking Loading Condition.

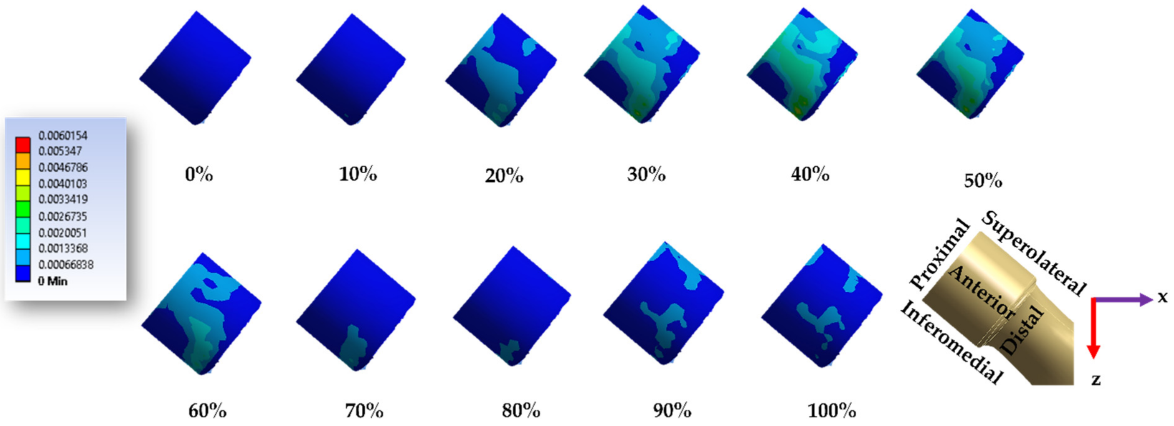

(a)

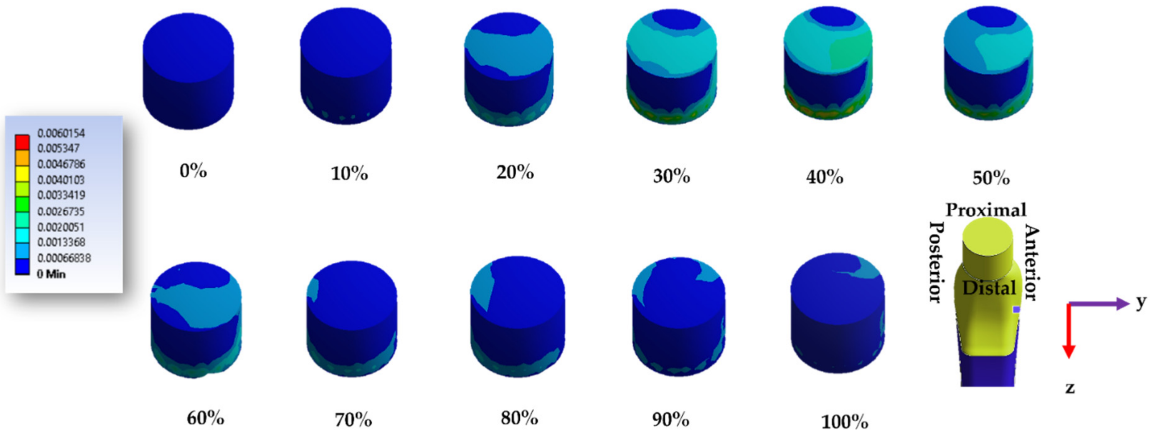

(b)

Figure S6. Sliding Distance Distribution Over Smooth Head-Neck Tapered Joint in, (a) Front and (b) Right Side View for Jogging Loading Condition.

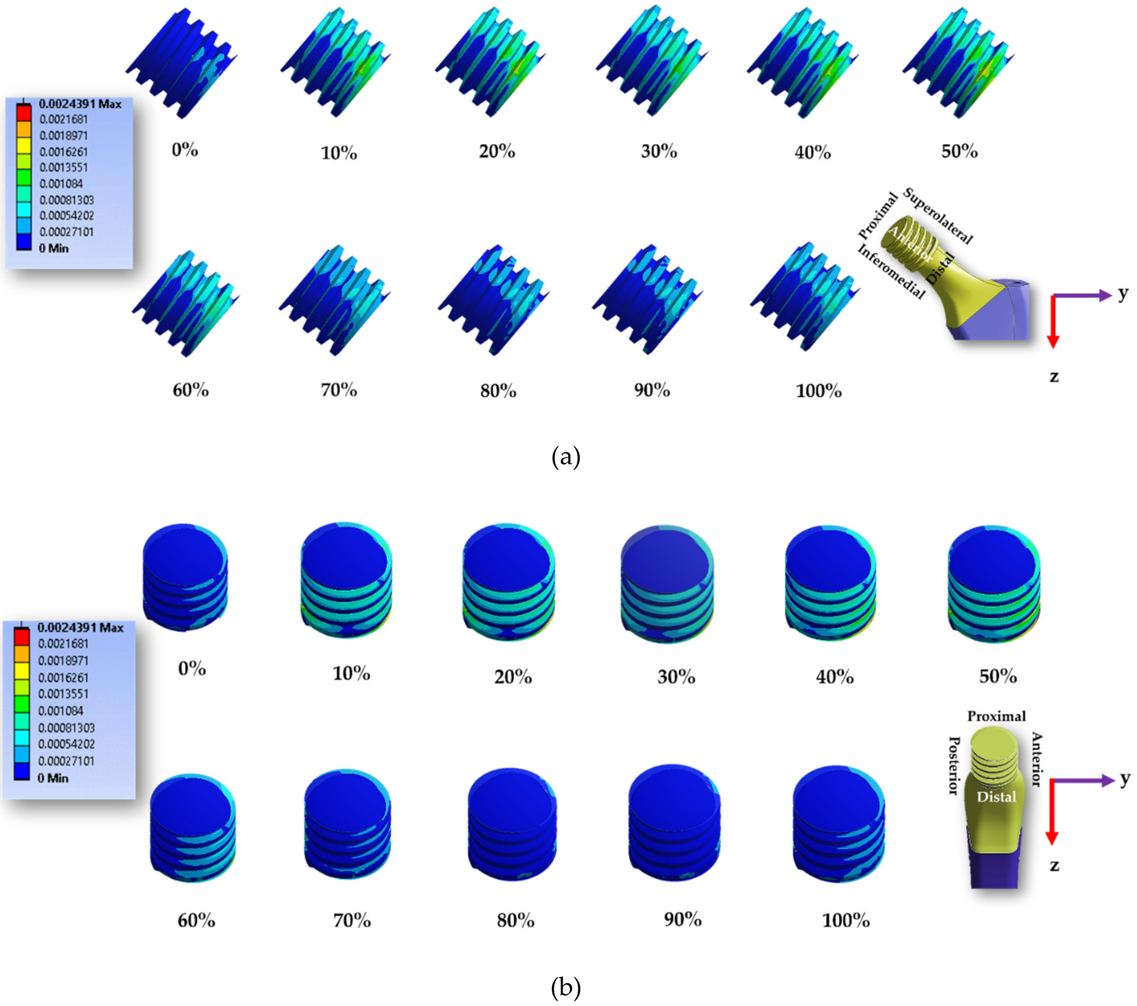

Figure S7. Sliding Distance Distribution Over Spiral Head-Neck Tapered Joint in, (a) Front and (b) Right Side View for Walking Loading Condition.

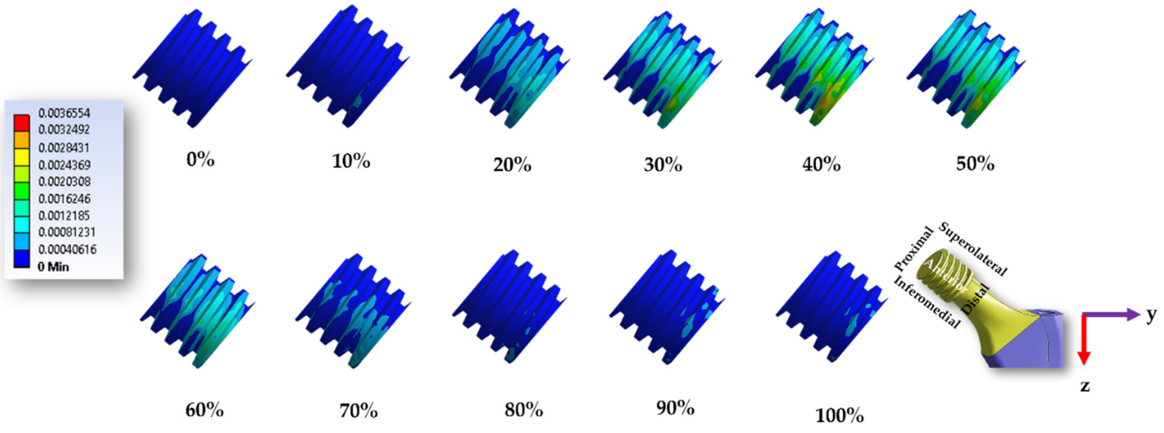

(a)

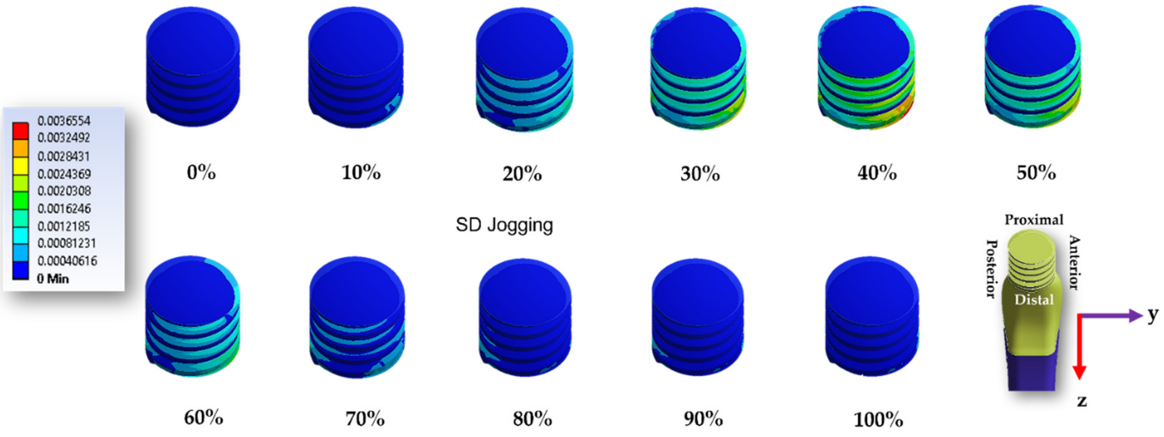

(b)

Figure S8. Sliding Distance Distribution Over Spiral Head-Neck Tapered Joint in, (a) Front and (b) Right Side View for Walking Loading Condition.
